# Diagnostic Accuracy of Chest Computed Tomography Scans for Suspected Patients With COVID-19: Receiver Operating Characteristic Curve Analysis

**DOI:** 10.2196/19424

**Published:** 2020-10-20

**Authors:** Lianpin Wu, Qike Jin, Jie Chen, Jiawei He, David M Brett-Major, Jianghu James Dong

**Affiliations:** 1 Department of Cardiology the Second Affiliated Hospital & Yuying Children’s Hospital of Wenzhou Medical University Wenzhou China; 2 College of Optometry Wenzhou Medical University Wenzhou China; 3 Department of Epidemiology University of Nebraska Medical Center Omaha, NE United States; 4 Department of Biostatistics and Department of Medicine University of Nebraska Medical Center Omaha, NE United States

**Keywords:** COVID-19, chest CT scans, nucleic acid testing, retrospective cohort study, AUC, ROC

## Abstract

**Background:**

Computed tomography (CT) scans are increasingly available in clinical care globally. They enable a rapid and detailed assessment of tissue and organ involvement in disease processes that are relevant to diagnosis and management, particularly in the context of the COVID-19 pandemic.

**Objective:**

The aim of this paper is to identify differences in the CT scan findings of patients who were COVID-19 positive (confirmed via nucleic acid testing) to patients who were confirmed COVID-19 negative.

**Methods:**

A retrospective cohort study was proposed to compare patient clinical characteristics and CT scan findings in suspected COVID-19 cases. A multivariable logistic model with LASSO (least absolute shrinkage and selection operator) selection for variables was used to identify the good predictors from all available predictors. The area under the curve (AUC) with 95% CI was calculated for each of the selected predictors and the combined selected key predictors based on receiver operating characteristic curve analysis.

**Results:**

A total of 94 (56%) patients were confirmed positive for COVID-19 from the suspected 167 patients. We found that elderly people were more likely to be infected with COVID-19. Among the 94 confirmed positive patients, 2 (2%) patients were admitted to an intensive care unit. No patients died during the study period. We found that the presence, distribution, and location of CT lesions were associated with the presence of COVID-19. White blood cell count, cough, and a travel history to Wuhan were also the top predictors for COVID-19. The overall AUC of these selected predictors is 0.97 (95% CI 0.93-1.00).

**Conclusions:**

Taken together with nucleic acid testing, we found that CT scans can allow for the rapid diagnosis of COVID-19. This study suggests that chest CT scans should be more broadly adopted along with nucleic acid testing in the initial assessment of suspected COVID-19 cases, especially for patients with nonspecific symptoms.

## Introduction

In December 2019, multiple cases of pneumonia were detected in Wuhan, Hubei Province, China [1]. These patients had nonspecific symptoms including variable fever, cough, breathing difficulties, muscle aches, and fatigue. Ultimately, this would prove to be the seed of a pandemic, named by the World Health Organization as COVID-19 [2], caused by the seventh member of the human-infecting coronavirus family, SARS-CoV-2. Other notable members in the same family are the severe acute respiratory syndrome (SARS) coronavirus and the Middle East respiratory syndrome (MERS) coronavirus, which have caused significant public health hazards. COVID-19 can be transmitted from person to person through droplets, contact, or through the fecal-oral route, with a high degree of communicability [3]. Since January 2020, COVID-19 has spread to most parts of China as well as the world, posing an extensive threat to global public health by seriously affecting the quality of life of millions of people. Large numbers of people across China were returning to their hometown from Wuhan for the Chinese Lunar New Year [4], making our region a COVID-19 epicenter. This retrospective study collected consecutively hospitalized patients with a suspected case of COVID-19 dating to the end of January 2020 at a single tertiary care referral hospital in Zhejiang Province. As of this writing, the epidemic in this region was well under control with few new cases and deaths from COVID-19.

In the context of this emergency and the need to move past nonspecific symptoms to approaches that allow for early diagnosis and management, we wanted to investigate potential differences in the chest CT scans of patients who were COVID-19 positive to those who were COVID-19 negative (confirmed via nucleic acid test). To demonstrate factors relevant to clinical outcomes, we wanted to compare patients with a confirmed positive case of COVID-19 to those with a negative result based on characteristics, interventions, and outcomes. This motivated us to propose a retrospective cohort approach for COVID-19 research. Several case-control studies have been carried out to investigate the clinical outcome of COVID-19. For example, several clinical characteristics have been identified in pregnant women and their neonates with COVID-19 [5]. Furthermore, COVID-19 is known to cause smell and taste disorders in those who are infected with the disease, particularly at a higher rate than patients with influenza [6]. However, there are few retrospective cohort studies that compare patients with confirmed COVID-19 to suspected cases through chest CT scans. Therefore, we conducted a retrospective cohort study to compare clinical characteristics and CT scan findings in suspected COVID-19 patients. Chest CT scans enable a rapid and detailed assessment of tissue and organ involvement in disease processes that are relevant to diagnosis and management, and so it is important to determine their usage for COVID-19, particularly since the outbreak of this coronavirus is seriously affecting people’s lives worldwide.

## Methods

### Data Source

In this study, consecutively hospitalized patients with a suspected case of COVID-19 from January 21, 2020, to February 11, 2020, were identified at a single tertiary care referral hospital and followed up to the end of April 2020. In the absence of neutrophilia as an indicator of an alternative diagnosis of bacterial infection, patients were classified as suspected COVID-19 cases. Due to isolation care practices, all suspected COVID-19 patients were assessed in the fever clinic of the hospital. All suspected COVID-19 patients were included in this cohort study. Afterward, all suspected COVID-19 patients underwent physical examinations, including routine blood tests, C-reactive protein testing, SARS-CoV-2 nucleic acid examination, and chest CT scans. If the chest CT scan findings of a patient were abnormal according to the physician, while the result of nucleic acid examination was negative, then the patient underwent another nucleic acid examination. Positive patients who were then confirmed to be infected with SARS-CoV-2 by nucleic acid testing were transferred to a special ward for care.

Chest CT scans were performed using a GE Light Speed VCT 64-slice and Phillips Brilliance 16-slice CT. The scanning parameters used were as follows: tube voltage of 120 kV, automatic tube current, scanning layer thickness of 5.00 mm, reconstruction layer thickness of 11.5 mm, image reconstruction by high-resolution algorithm, and a matrix with 512 × 512 = 262,144 pixels. The lung window had a position of 500 Hounsfield units (HU) and a wider window width of 1500 HU. The mediastinum window had a position of 40 HU and a window width of 400 HU. Chest CT scans were focused on observing the density, number, distribution, location, and morphology of the lesions, as well as pleural thickening, pleural effusion, mediastinal lymph node enlargement, or other accompanying signs. In the early stages, one or two lungs had many shadows, and there were multiple ground glass density shadows. The internal lung texture was grid-like with halo signs around; the long axis of some lesions was parallel to the pleura and was not distributed according to lung segments. There was no cavity formation, no pleural effusion, and no significant swelling of mediastinal lymph nodes. There were also signs of bronchial ventilation. As the disease progresses, the diseased area rapidly increased and expanded, advancing from the periphery to the center along the bronchial vascular bundle, and may also be distributed in the form of antibutterfly wings. Therefore, we selected the following 12 CT indicators to better understand the imaging characteristics and severity of COVID-19: (1) location of lesions (ie, the left, right, or both lungs); (2) number of lesions; (3) location of the lesions along with the bronchus beam distribution, near pleural distribution, or mixed distribution; (4) lesion(s) size; (5) presence of the air bronchi sign; (6) presence of grid-like texture; (7) lesion morphology; (8) percentage of lung involvement; (9) presence of atelectasis; (10) whether the density of the lesion is ground-glass–like, solid, or mixed; (11) presence of pleural effusion, lymphadenopathy, or extrapulmonary manifestations; and (12) other potential lung disease features such as pulmonary bullae, pulmonary nodules, calcifications, and fibrous lesions.

### Study Design and Statistical Analysis

We divided all hospitalized patients with suspected COVID-19 in the cohort into either the positive group or the negative group based on the results of SARS-CoV-2 nucleic acid testing. One group was COVID-19 positive and the other group was COVID-19 negative. We wanted to compared patient epidemiological characteristics as well as clinical laboratory and CT scan findings in these two groups.

We summarized continuous variables as either means and standard deviations or medians with interquartile ranges for all patients in both the positive and negative groups. For categorical variables, we calculated the percentages of patients in each category. Characteristics for case and control patients were compared using the Student *t* test and analysis of variance, or nonparametric statistics (rank-sum tests) and chi-square tests as appropriate. An alpha of .05 was used as the cutoff for statistical significance. A LASSO (least absolute shrinkage and selection operator) logistic model for variable selection was used to identify the key predictors from all available variables. The area under the curve (AUC) with 95% CIs were calculated for each of the selected predictors, and the overall AUC of all these predictors was provided to show the most accurate combined variables for the biomarkers of COVID-19 from all available variables in the study. All analyses were done with SAS software, version 9.4 (SAS Institute Inc).

### Ethics Statement

This was a retrospective case series study, and no patients were involved in the study design. Therefore, consent was waived. Ethics approval for this project was obtained from the Institutional Review Board of the University of Nebraska Medical Center (IRB #1982933).

## Results

### Patient Epidemiological Characteristics

A total of 167 patients with a suspected case of COVID-19 were identified and included in the statistical analysis. Their basic characteristics are shown in Table 1. Patients were aged 1-87 years with a mean age of 44 (SD 19) years; 92 (55%) were men and 75 (45%) were women. Of these initial 167 suspected COVID-19 cases, 94 (56%) were confirmed positive, and 38 (23%) had a history of traveling to Wuhan. Comorbidities varied; 23 (14%) patients had hypertension, 13 (8%) had diabetes, 13 (8%) had cardiogenic diseases, less than 5 (1%) had lung disease, less than 5 (1%) had kidney disease, and less than 5 (1%) had liver disease. Among 123 (74%) patients who exhibited symptoms, the most common symptoms were fever (n=123, 74%), cough (n=105, 63%), runny nose (n=22, 13%), gastrointestinal symptoms (n=22, 13%), sore throat (n=42, 25%), fatigue (n=32, 19%), and muscle pain (n=31, 19%). In total, 43 (26%) patients had been treated by Chinese medicine, which includes Lianhua Qingwen capsules and Jinhua Qinggan granules. Among the 94 patients who were confirmed COVID-19 positive, 2 (2%) patients were admitted to an intensive care unit (ICU) during this study. Both ICU patients were elderly individuals with hypertension or diabetes. No patients died during the study period.

**Table 1:** Epidemiological characteristics of suspected cases of COVID-19.

Characteristic		Total (N=167)	COVID-19 positive (n=94)	COVID-19 negative (n=73)	*P* value
Age (years), mean (SD)		44 (19)	47 (14)	38 (23)	
**Age category (years) (%)**					<.001
	<18	11	1	23	
	18-39	27	24	30	
	40-59	45	57	29	
	≥60	17	18	18	
Male (%)		55	60	50	.22
Travel history to Wuhan (%)		23	28	17	.04
**Comorbidities (%)**					
	High blood pressure	14	16	13	.79
	Diabetes	8	12	6	.26
	Cardiogenic diseases	8	6	8	.88
	Lung disease	1	1	0	N/A^a^
	Anemic	0	0	0	N/A
	Stroke	1	1	0	N/A
	Kidney disease	0	0	0	N/A
	Surgery	11	13	7	.94
	Liver disease	0	0	0	N/A
**First symptoms at admission (%)**					
	Fever	74	74	75	.65
	Cough	63	73	55	.61
	Runny nose	13	9	20	.23
	Gastrointestinal symptoms	13	14	32	>.99
	Sore throat	25	19	25	.48
	Fatigue	19	18	18	>.99
	Muscle pain	19	26	14	.08
Body temperature, mean (SD)		37.18 (0.79)	37.13 (0.82)	37.27 (0.78)	.07
Pulse, mean (SD)		90 (19)	84 (14)	92 (20)	.003
Respiratory rate, mean (SD)		19.72 (2.47)	19.13 (1.77)	20.25 (3.27)	.005
Blood pressure, mean (SD)		42 (2.13)	47 (14)	38 (23)	<.001
**Medicine during the hospital (%)**					
	Antivirus	73	75	71	.64
	Antibiotics	55	45	68	.002
	Hormone	13	22	0	<.001
	Chinese medicine^b^	26	47	0	<.001
	Intensive care unit	1	2	0	N/A

^a^N/A: not applicable.

^b^Refers to Lianhua Qingwen capsules and Jinhua Qinggan granules.

### Biochemical Testing

The results of clinical laboratory testing are summarized in Table 2. C-reactive protein levels of the confirmed positive patients (median 8.25, IQR 1.57-15.40) were significantly higher than those of confirmed negative patients (median 6.75, IQR 1.07-30.19). White blood cell count (median 4.89, IQR 4.05-5.68) was significantly lower in confirmed positive cases compared to confirmed negative ones (median 7.99, IQR 5.08-10.03). Similarly, for red blood cell count, the confirmed positive group had a significantly lower value (median 4.49, IQR 4.03-4.90). Hemoglobin levels (median 134.20, IQR 108.11-149.23) of confirmed positive patients was significantly lower than confirmed negative patients (median 139.00, IQR 114.00-150.00).

**Table 2:** The results of routine blood tests from the clinical laboratory.

Routine blood tests	Total (N=167)	COVID-19 positive (n=94)	COVID-19 negative (n=73)	*P* value
C-reactive protein (mg/L), median (IQR)	7.10 (1.30-17.50)	8.25 (1.57-15.40)	6.75 (1.07-30.19)	<.001
White blood cell count (10^9^/L), median (IQR)	5.38 (4.45-7.67)	4.89 (4.05-5.68)	7.99 (5.08-10.03)	<.001
Red blood cell count (10^9^/L), median (IQR)	4.65 (4.09-5.00)	4.49 (4.03-4.90)	4.78 (4.40-5.08)	.002
Lymphocyte count (10^9^/L), median (IQR)	1.16 (0.93-1.56)	1.30 (0.96-1.51)	1.11 (0.77-1.66)	.10
Hemoglobin (g/L), median (IQR)	137.24 (109.00-149.00)	134.20 (108.11-149.23)	139.00 (114.00-150.00)	.86
Platelet count (10^9^/L), median (IQR)	142.00 (59.00-185,00)	145.1 (65.21-180.23)	134.00 (46.00-198.00)	.17
Plasma prothrombin time determination, median (IQR)	13.65 (13.00-13.90)	13.50 (13.10-13.81)	13.80 (10.80-15.15)	<.001
Thrombin time (second), median (IQR)	15.21 (14.45-15.76)	15.10 (14.43-15.70)	15.50 (14.50-16.10)	.54
Activated partial thromboplastin (second), median (IQR)	41.70 (40.00-45.80)	43.46 (40.10-45.60)	41.30 (38.00-46.80)	.003
D-D dimer (μg/ml), median (IQR)	0.45 (0.30-0.70)	0.40 (0.08-0.60)	0.70 (0.44-1.03)	.42
Alanine aminotransferase (IU/L), median (IQR)	28.70 (8.00-45.00)	33.00 (7.00-40.00)	25.50 (8.00-53.00)	.02
Urea nitrogen (mmol/L), median (IQR)	3.97 (3.39-4.90)	4.03 (3.43-4.66)	3.76 (3.15-6.19)	.19
Fibrinogen (g/L), median (IQR)	4.90 (4.06-5.67)	4.88 (4.07-5.67)	5.03 (3.31-5.90)	.69
Eosinophil count, median (IQR)	0.03 (0.01-0.09)	0.02 (0.01-0.07)	0.04 (0.01-0.11)	<.001
Hematocrit, median (IQR)	0.40 (0.37-0.44)	0.39 (0.36-0.42)	0.41 (0.38-0.45)	.005

### Chest CT Scans and Imaging

In the first chest CT scan of the 167 patients with a suspected case of COVID-19, 80 (48%) of the confirmed positive patients had lesions in both of their lungs compared to 27 (30%) of the confirmed negative patients. Additionally, 73 (44%) patients in the positive group had more than three lesions compared to 28 (17%) patients in the negative group. Among the 94 confirmed positive cases, 89 (95%) had patch lesion morphology. The detailed comparative results are summarized in Table 3.

**Figure 1:**
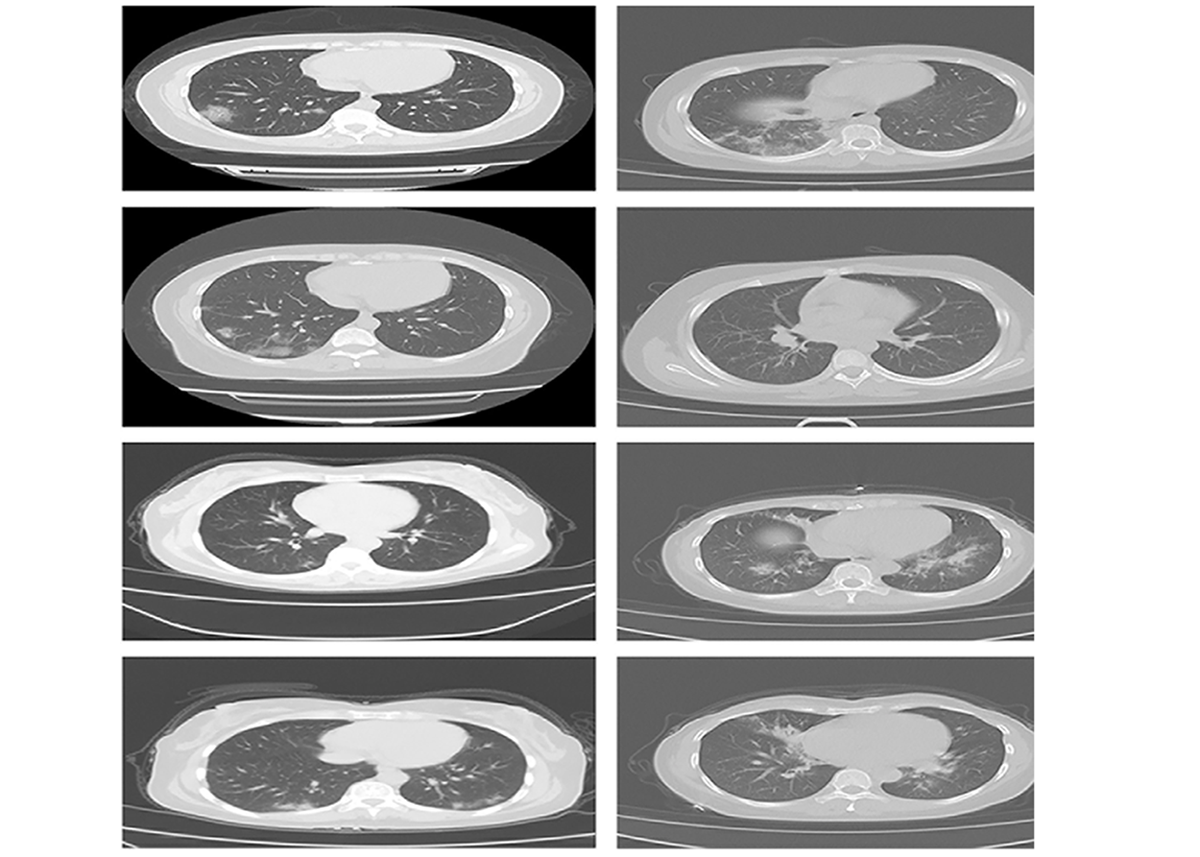
The images in the left panel are CT scans of four randomly selected confirmed COVID-19–positive patients. There are multiple patchy lesions, and the grinding glass shadows are distributed in the peripheral pulmonary field. The images in the right panel are CT scans of four randomly selected suspected patients who were confirmed to be COVID-19 negative. Their lesions are large, strip-like, and have uneven density. Pleural thickening near affected lung segments is also seen.

CT scans of 8 randomly selected patients are shown in Figure 1. We found substantial, reliable differences in the chest CT scans of patients with COVID-19 from those who were suspected cases confirmed negative via nucleic acid testing. The dominant phenotype on the CT scan was the presence of multiple patchy lesions and a distribution of ground glass shadows in the peripheral pulmonary field, where denser lesions were large and strip-like with uneven density. Pleural thickening near affected lung segments was also seen in these patients.

**Table 3:** Chest computed tomography (CT) results with lesions and imaging manifestations.

CT image feature		COVID-19 positive (n=94)	COVID-19 negative (n=73)	*P* value
**Distribution of lesions (%)**				<.001
	No lesions	0	1	
	Left lung	6	27	
	Right lung	9	42	
	Both left and right lungs	85	30	
**Number of lesions (%)**				<.001
	1	9	46	
	2	9	16	
	≥3	81	38	
**Locations of lesions (%)**				.01
	Distributed along the bronchogram	11	15	
	Close to the pleura	50	30	
	Mixed distribution	39	55	
**Size of lesion (cm) (%)**				<.001
	<1	11	37	
	1-3	26	28	
	>3	63	34	
**Air bronchogram sign (%)**				<.001
	Yes	17	11	
	No	83	89	
**Lesions’ internal texture (%)**				<.001
	Lattice texture	28	22	
	No lattice texture	72	78	
**Lesion morphology (%)**				.003
	Patch	95	37	
	Pulmonary segments	2	26	
	Lobe involvement	3	37	
**Lung involvement (%)**				<.001
	<25%	59	73	
	50%-75%	28	22	
	>75%	16	5	
**Atelectasis (%)**				<.001
	Yes	2	8	
	No	98	91	
**Lesion density (%)**				.001
	None	3	1	
	Ground glass	23	19	
	Solid	3	29	
	Mixed	71	51	
**Extrapulmonary manifestations (%)**				<.001
	Pleural effusion	3	1	
	Lymphadenopathy	3	12	
	None	94	87	
**Other lung diseases including bullae, pulmonary nodule, fibrous stove, calcification, and tuberculosis (%)**				<.001
	Yes	33	22	
	No	67	78	

### Multivariable Analysis

**Figure 2:**
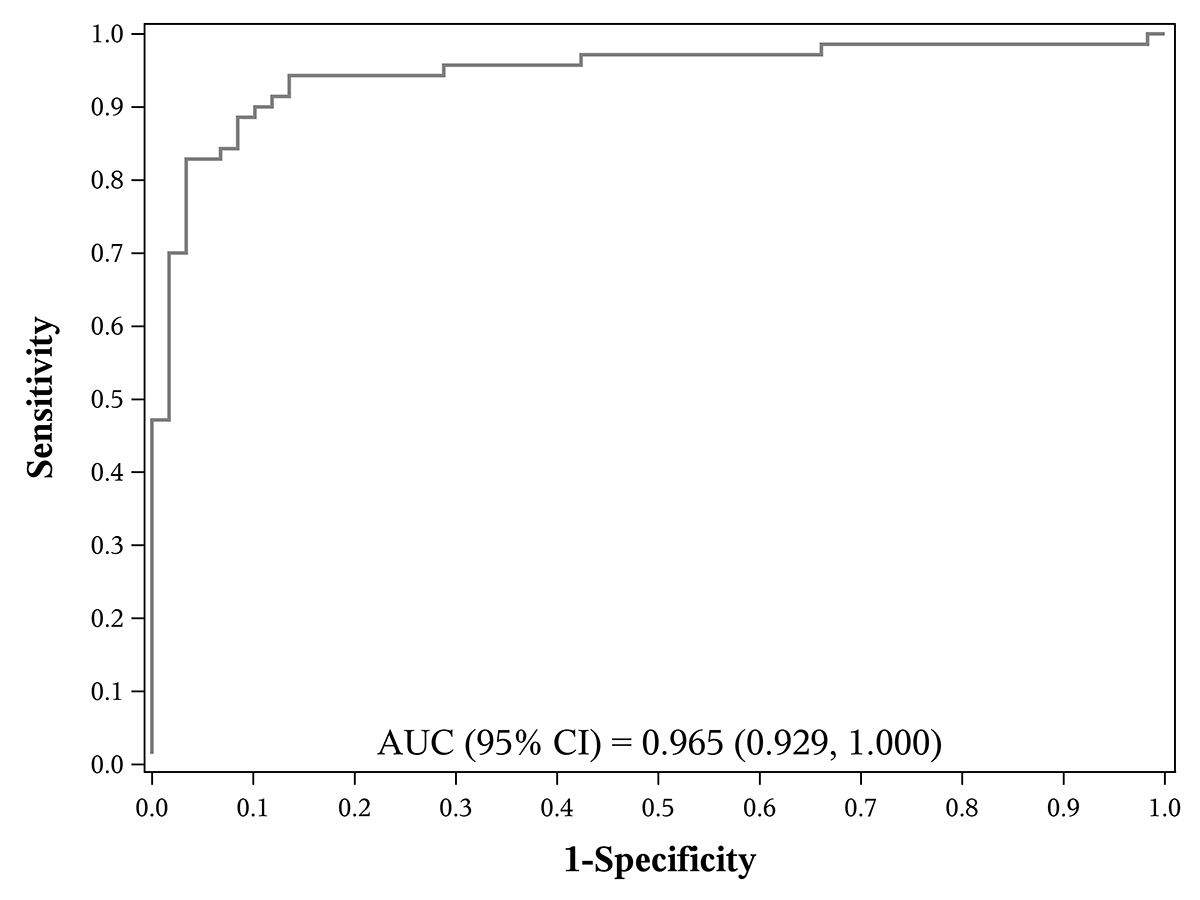
The receiver operating characteristic (ROC) curve of selected variables for COVID-19–positive cases. The ROC curve demonstrates the trade-off between sensitivity and specificity, and shows that the biomarker with a combination of selected variables is the most accurate test for identifying patients with COVID-19 due to an area under the curve (AUC) value of 0.97 (95% CI 0.93-1.00).

A multivariable LASSO logistic model for variable selection was used to identify the key predictors from all variables in Tables 1-3. We found the key predictors for COVID-19 to be white blood cell count, lesion morphology, distribution of lesions, cough, locations of lesions, and travel history to Wuhan. The AUC for each of the selected predictors ranged from 0.56 to 0.80, as shown in Table 4. The overall AUC of all these selected key predictors was 0.97 (95% CI 0.93-1.00), as shown in Figure 2.

**Table 4:** Area under the curve (AUC) values for the selected predictors from the logistic model with LASSO (least absolute shrinkage and selection operator) selection.

Selected variables	AUC (95% CI)
White blood cell count	0.80 (0.72-0.87)
Lesion morphology	0.78 (0.71-0.84)
Distribution of lesions	0.76 (0.69-0.84)
Cough	0.59 (0.52-0.66)
Locations of lesions	0.56 (0.47-0.65)
Travel history to Wuhan	0.55 (0.48-0.61)
Overall AUC of the above selected variables	0.97 (0.93-1.00)

## Discussion

This retrospective cohort study described the differences in clinical characteristics between patients who were confirmed COVID-19 positive to those confirmed to be COVID-19 negative. Elderly people were more likely to be infected by COVID-19. We also found significant associations between low white blood cell count, high C-reactive protein, and a subsequent positive COVID-19 test. Regarding the low white blood count, this is common in viral infections and is of a larger magnitude than the absence of leukocytosis. Fever symptoms are the first clinical characteristics, alongside gastrointestinal reactions, sore throat, fatigue, and muscle pain. However, many patients (n=24, 26%) did not have a fever and cough. Of the original 167 suspected patients, 6 (4%) were negative according to the results of their first nucleic acid test, but they were consequently confirmed positive after performing CT chest scans. Chest CT scans can supply a better understanding of COVID-19, especially for patients with no specific symptoms, such as fever and cough.

While other studies have begun to describe clinical experiences of this novel disease, our patients had not yet been described nor this question addressed [7]. For example, laboratory examination showed normal or decreased peripheral white blood cells, and CT scan findings showed that the locations of lesions are more likely to be close to the pleura. Compared with the clinical outcomes of other studies [8-11], fewer patients (less than 5%) were admitted to an intensive care unit in our study, and no patients died during the study period. More importantly, few studies have provided a comparison of patients who were confirmed COVID-19 positive to those who were confirmed COVID-19 negative. Therefore, one strength of this paper is that we can clearly show the differences between confirmed positive and confirmed negative patients through our retrospective cohort design. The comparative results can provide some valuable information in the understanding of the clinical features of COVID-19 to help identify patients who have the coronavirus from a large group of patients who may or may not be infected. This is especially applicable to current times since the number of people suspected to be infected with the disease is increasing rapidly worldwide. Another strength of this paper is that we could identify the top key predictors from all predictors based on the multivariable LASSO logistic model. The area under the multivariable receiver operating characteristic (ROC) curve (0.97, 95% CI 0.93-1.00) illustrates that the combined biomarker of these selected key predictors is a good index for COVID-19. However, this study has some inherent limitations as a retrospective cohort study. For example, we did not have additional laboratory or lung function data to measure immune responses. This retrospective study lacks a formal sample size and power calculations. The power of this study may be limited due to the relatively small number of infected patients with chest CT scans; the study also took place at a single tertiary care referral hospital. The results need to be replicated with a larger number of COVID-19–confirmed cases in future studies.

With our well-characterized patients, this retrospective cohort study can enhance our understanding of the use of chest CT scans for COVID-19 case management. For example, we found that the early signs of CT lesions are in both lungs, and locations of lesions are more likely to be close to the pleura. We also observed that the lesion distribution gradually expands from the periphery to the center, and the lesion density is more inclined to be mixed. Therefore, chest CT scans should be more broadly adopted along with nucleic acid testing in the initial assessment of suspected COVID-19 cases when the nucleic acid examination is delayed or negative, or when further clinical characterization of severity is needed, especially for patients with nonspecific symptoms.

## Abbreviations

AUC: area under the curve
CT: computed tomography
HU: Hounsfield unit
ICU: intensive care unit
LASSO: least absolute shrinkage and selection operator
MERS: Middle East respiratory syndrome
ROC: receiver operating characteristic
SARS: severe acute respiratory syndrome
