# Correction：Diagnostic Accuracy of Chest Computed Tomography Scans for Suspected Patients With COVID-19: Receiver Operating Characteristic Curve Analysis

**DOI:** 10.2196/25829

**Published:** 2020-11-20

**Authors:** Lianpin Wu, Qike Jin, Jie Chen, Jiawei He, David M Brett-Major, Jianghu James Dong

**Affiliations:** 1 Department of Cardiology the Second Affiliated Hospital & Yuying Children’s Hospital of Wenzhou Medical University Wenzhou China; 2 College of Optometry Wenzhou Medical University Wenzhou China; 3 Department of Epidemiology University of Nebraska Medical Center Omaha, NE United States; 4 Department of Biostatistics and Department of Medicine University of Nebraska Medical Center Omaha, NE United States

In “Diagnostic Accuracy of Chest Computed Tomography Scans for Suspected Patients With COVID-19: Receiver Operating Characteristic Curve Analysis” (JMIR Public Health Surveill 2020;6(4):e19424), the authors noted one error.

The affiliation for authors Lianpin Wu, Qike Jin, and Jiawei He was incorrectly listed as:

Department of Cardiology, Wenzhou Medical University, Wenzhou, China

The affiliation for these authors has been corrected to:

Department of Cardiology, the Second Affiliated Hospital & Yuying Children’s Hospital of Wenzhou Medical University, Wenzhou, China

The correction will appear in the online version of the paper on the JMIR Publications website on November 20, 2020, together with the publication of this correction notice. Because this was made after submission to PubMed, PubMed Central, and other full-text repositories, the corrected article has also been resubmitted to those repositories.

